# A repeated strike loading organ culture model for studying compression-associated chronic disc degeneration

**DOI:** 10.17305/bb.2024.10640

**Published:** 2024-08-04

**Authors:** Baoliang Li, Xu Chen, Hongkun Chen, Fu Zhang, Jianfeng Li, Zhengya Zhu, Tao Tang, Manman Gao, Nianhu Li, Liang Ma, Zhiyu Zhou

**Affiliations:** 1 Department of Orthopaedics, Affiliated Hospital of Shandong University of Traditional Chinese Medicine, Jinan, China; 2 Innovation Platform of Regeneration and Repair of Spinal Cord and Nerve Injury, Department of Orthopaedic Surgery, The Seventh Affiliated Hospital, Sun Yat-sen University, Shenzhen, China; 3 Department of Orthopaedic Surgery, The Affiliated Hospital of Xuzhou Medical University, Xuzhou, China; 4 Department of Orthopaedics, The Second Affiliated Hospital of Anhui Medical University, Hefei, China; 5 Guangdong Provincial Key Laboratory of Orthopedics and Traumatology, Orthopaedic Research Institute/Department of Spinal Surgery, The First Affiliated Hospital of Sun Yat-sen University, Guangzhou, China; 6 Department of Sport Medicine, Institute of Translational Medicine The First Affiliated Hospital of Shenzhen University, Shenzhen Second People’s Hospital, Shenzhen, China; 7 Shenzhen Key Laboratory of Anti-aging and Regenerative Medicine, Department of Medical Cell Biology and Genetics, Health Sciences Center Shenzhen University, Shenzhen, China

**Keywords:** Intervertebral disc degeneration (IDD), mechanical stress, organ culture model

## Abstract

Mechanical stress has been viewed as one of the key risk factors in accelerating the intervertebral disc degeneration (IDD) process. The goal of the present study was to employ a repeated strike loading bovine caudal disc system to elucidate the pathophysiological impacts of cumulative mechanical stress on the disc. The discs in the model groups were subjected to two different mechanical stresses: one strike loading or repeated strike loading. The following indices were analyzed: histological morphology, glycosaminoglycan release, disc height, cell viability, apoptosis-related protein expression, and catabolism-related gene expression. Both mechanical stress modes induced degenerative changes in the discs by day 11, such as clefts and delamination of the annulus fibrosus (AF); they increased glycosaminoglycan release. Cell viability was significantly decreased and catabolic gene expression was significantly upregulated in the degenerative loading group and repeated strike loading group by day 9. These alterations remained evident in the AF tissue of the repeated strike loading group on day 11. Our data suggests that the repeated strike loading model adopted in this study could lead to degenerative changes in the disc organ model. AF cells displayed a more noticeable response to mechanical stress damage and a slower recovery process, suggesting that AF serves as a pivotal factor in disc degeneration due to mechanical stress injuries. The study also indicates that due to the gradual self-repair of intervertebral disc (IVD) cells after injury, it is necessary to apply repeated strike loading on the disc at specific intervals when researching the repair of chronic disc injuries.

## Introduction

Intervertebral disc degeneration (IDD) is one of the primary contributing factors of low back pain, which causes a substantial socioeconomic burden worldwide [1]. In Western countries, approximately 80% of the population is affected by low back pain, with its incidence rising with advancing age [2]. Acute pain from disc degeneration costs up to $100 billion annually in the U.S. [3]. Though significant progress has been made in exploring the pathogenesis of IDD, the molecular mechanisms still hold some ambiguity.

The intervertebral disc (IVD) is an avascular organ positioned between neighboring vertebral bodies and is responsible for load transmission during daily activity. It is composed of three fundamental tissues: the gelatinous inner nucleus pulposus (NP), which is encircled by the annulus fibrosus (AF), and interposed between two cartilaginous endplates (CEPs) [4]. The CEP is a layer of hyaline cartilage that is crucial for the nutrient and metabolite exchange between the IVD and vertebral body [5]. The NP contains large amounts of proteoglycans and glycosaminoglycans (GAGs), with high hydration characteristics, allowing for the conversion of compressive pressure into evenly distributed expansion pressure. Studies have shown that the degeneration of NP tissue reduces the disc’s ability to absorb and distribute loads evenly across the vertebral column [6]. This alteration in mechanical properties leads to increased mechanical stress on other components of the disc, accelerating the degeneration process [7]. The AF is rich in highly organized type I collagen bundles that are ideal for generating tensile strength to balance the expansion pressure from the NP [8]. Degeneration of the AF leads to a decrease in its structural integrity. This deterioration can cause the AF to become more susceptible to tears and fissures, allowing the NP material to herniate or extrude, contributing to spinal instability and pain [9].

A vast body of research has been devoted to the study of IVD degeneration. The selection of an appropriate experimental model is crucial; both cell and animal models are instrumental yet present notable limitations. Primarily, these models fail to fully encapsulate the complex and multifaceted nature of human IDD pathophysiology due to significant differences in cell population, tissue composition, and biomechanical properties across species [10]. Furthermore, induced degenerative changes in animal models may not accurately reflect the human condition, limiting their applicability and the translation of findings to clinical scenarios [11]. Consequently, while these models contribute valuable insights into disc biology, caution must be exercised when extrapolating results to human IDD treatment and prevention strategies. Given these considerations, the use of IVD organ culture has been identified as a compelling method to mitigate the limitations inherent in both in vivo and in vitro models. Bovine caudal discs have been recognized for their pronounced similarities to mature human discs in critical areas including cellular composition, microstructural mechanics, and extracellular matrix composition [12, 13]. These similarities render bovine caudal discs an invaluable model for preclinical research, facilitating studies that are both relevant and translatable to human conditions.

The IVD is subjected to diverse physiological and pathological mechanical stresses, including compressive dynamic/static loads, tensile strains, and osmotic stress. Previous epidemiological and clinical research has identified strike loading as a significant contributor to the initiation and progression of IDD [14]. Repeated mild lumbar injury is not uncommon in our daily lives, especially for those who perform manual work and athletes. Epidemiological evidence has pointed out that repeated lumbar injury exposure is an important risk factor for IDD [15, 16]. Additionally, a recent study found that progressive degenerative changes in rabbit lumbar discs were induced by repeated axial loading [17]. An animal model of accelerated disc degeneration demonstrated that repeated stab injury initiated a vicious cycle of inflammation and matrix damage [18]. We have proven in a strike-loading disc degeneration model that a short-term degeneration reaction is initiated after one strike loading. This reaction is self-limited and typically ceases within a few days [19]. While most studies have focused on exploring the timely pathophysiological consequences of mechanical stress on the IVD, few studies have investigated whether cumulative mechanical stress could induce a persistent molecular response in the IVD.

To better clarify the relationship between repeated acute strike loading and IDD, we employed a fully developed IVD organ culture system in this study. Specifically, the deleterious effects of one strike loading and repeated strike loading on the IVD were assessed. We hypothesized that repeated strike loading would result in persistent IVD degeneration.

## Materials and methods

### Separation and nurture of IVDs

Bovine tails were obtained from 18-month-old cows within 6 h of slaughter. Since we used residues from an abattoir, there was no need for ethical approval. IVDs were harvested using the method described earlier on Day 0 [20]. In short, the peripheral tissues were dissected with a scalpel blade, and then IVDs along with the endplates were separated using a sawing machine. The IVDs underwent cleaning with an irrigator with PBS and then were washed with PBS and 10% penicillin/streptomycin (Gibco) in an orbital shaker at 37 ^∘^C for 15 min. IVDs were cultured at 37 ^∘^C in a 5% CO_2_ incubator, and the medium was changed daily. The culture medium was composed of Dulbecco’s modified Eagle’s medium (Sigma–Aldrich) supplemented with 10% fetal bovine serum, 1% penicillin/streptomycin, 1% ITS+1 (Sigma–Aldrich), 50 µg/mL L-ascorbic acid (Sigma–Aldrich), and 0.1% Primocin.

### Strike loading application

**Figure 1.**
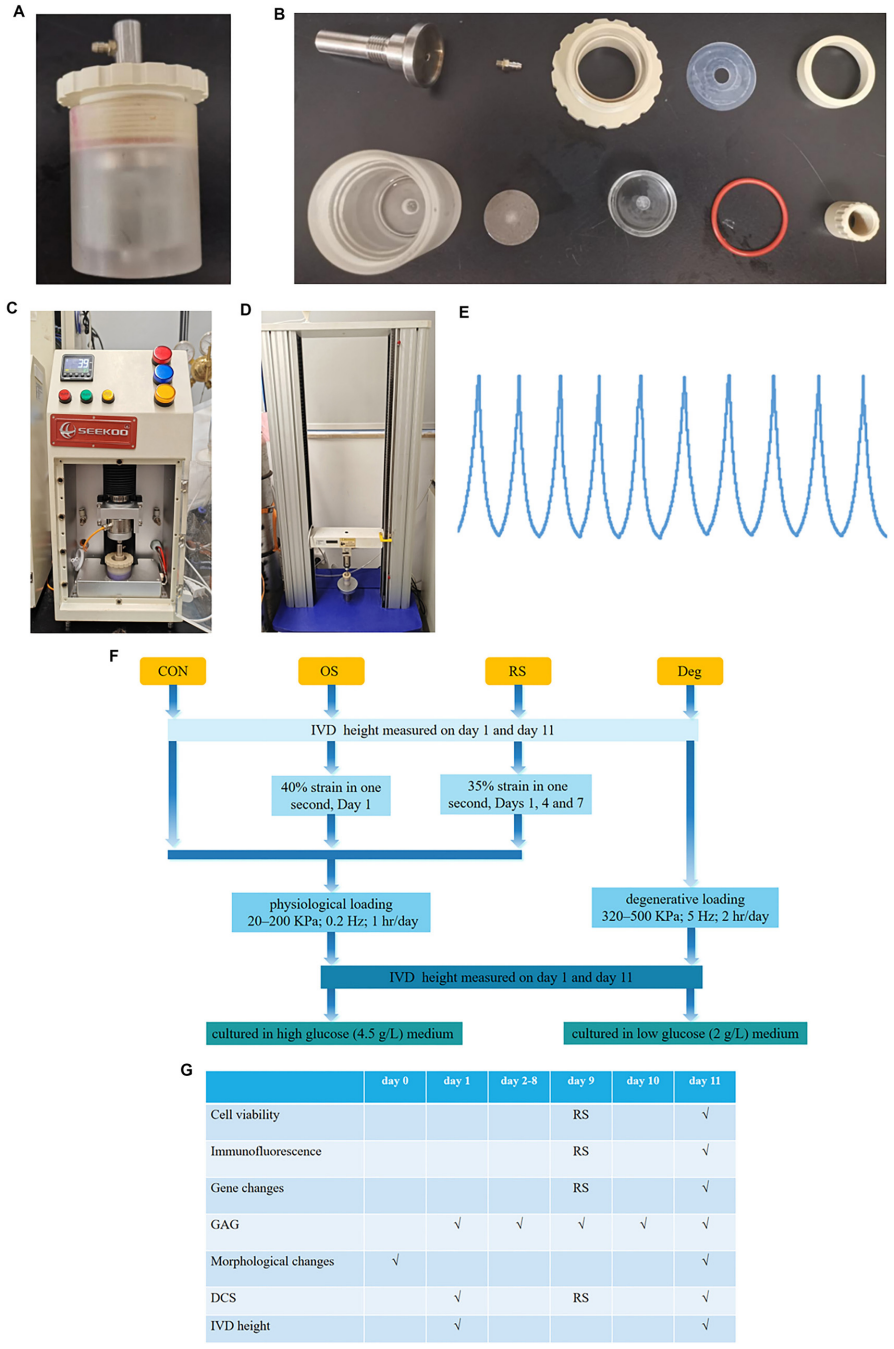
(A) Customized chamber; (B) The parts of the customized chamber; (C) Custom-designed bioreactor; (D) Specially designed mechanical testing machine; (E) Representative sinusoidal stress curves of the IVDs’ physical loading pattern using the custom-designed bioreactor; (F) Flowchart of grouping IVDs into different models; (G) The culture time and the outcome parameters. CON: Control group; OS: One strike group; RS: Repeated strike group; Deg: Degenerative group; IVD: Intervertebral disc.

IVDs from the same animal were randomized into four groups: control (CON) group, degenerative (Deg) group, one strike (OS) group, and repeated strike (RS) group. To maintain cell viability, IVDs in the CON and strike groups underwent physiological sinusoidal loading (20–200 KPa; 0.2 Hz; 1 h/day) in our customized chamber (Figure 1A) with fresh high-glucose (4.5 g/L) medium using our custom-designed bioreactor (Figure 1C), and then were cultured in fresh high-glucose medium overnight. IVDs in the Deg group underwent degenerative sinusoidal loading (320–500 KPa; 5 Hz; 2 h/day) in our customized chamber with fresh low-glucose (2 g/L) medium using our custom-designed bioreactor, and then were cultured in fresh low-glucose medium overnight [20]. IVDs in the OS group were only subjected to strike loading (40% strain in one second) on day 1. IVDs in the RS group were subjected to strike loading (35% strain in one second) on days 1, 4, and 7. The IVDs underwent an initial 3-min preloading of 10 N to prevent over-hydration and to ensure proper connection among the IVD, the customized chamber, and the specially designed mechanical testing machine (Figure 1D). Then the mechanical testing machine was employed to subject the IVD to strike loading for a duration of 1 s, inducing strains equivalent to 40% or 35% of the IVD [7]. IVDs in the CON group, Deg group, and OS group were collected on Day 11 for further analysis. IVDs in the RS group were collected on days 9 and 11.

**Table 1:** Primers used for qPCR

**Gene**	**Gene ID**	**Primers**	**Size of amplicon (bp)**
*ADAMTS4*	286806	F:5′-TACCGAGGGACTGAACTCCACATC-3′ R:5′-GGAATGCCGCCATCTTGTCATCT-3′	238
*ADAMTS5*	286805	F:5′-TGTGCGGTGATTGAAGACGATGG-3′ R:5′-TGCTGGTGAGGATGGAAGACATTAAG-3′	157
*MMP1*	281308	F:5′-CCAGACCTGTCAAGAGCAGATGT-3′ R:5′-ATGAGCGTCTCCTCCGATACCT-3′	222
*MMP3*	281309	F:5′-AACCTTCCGATTCTGCTGTTGCTA-3′ R:5′-GCTTGCGTATCACCTCCAGAGT-3′	262
*COL2*	407142	F:5′-GAGCAGCAAGAGCAAGGACAAGA-3′ R:5′-GCAGTGGTAGGTGATGTTCTGAGAG-3′	169
*IL-6*	280826	F:5′-TGATGAGTGTGAAAGCAGCAAGGA-3′ R:5′-TCTTCTCCAGCAGGTCAGTGTTTG-3′	299
*RPLP0*	286868	F:5′-CACGCTGCTGAACATGCTGAAC-3′ R:5′-AGGCACACGCTGGCAACATT-3′	165

### Cell viability analysis

After the dissection of one endplate, whole IVDs were snap-frozen in optimal cutting temperature compound (Sakura). Ten-micrometer transverse cryosections were cut with a microtome (Leica). Cell viability was measured by double labeling the cells with lactate dehydrogenase (LDH, cells stained blue) and ethidium homodimer-1 (EthD-1, nuclei emitted red fluorescence) [21]. Cell viability was assessed in the NP and AF. Images were visualized at 20× magnification using a DM6B fluorescence microscope (Leica). Pure red fluorescence represents dead cells, while blue or blue/red fluorescence represents live cells. Image-J software was employed to count the living and dead cells as follows: briefly, click on each cell to mark it with a point, each click will place a mark and keep a count.

### Immunofluorescence

IVD tissue was fixed in fresh 4% paraformaldehyde overnight before dehydration in graded sucrose solutions and embedding in an OCT mounting medium. 10 um thick sections were cut with a microtome (Leica). The following antibodies were used: CASPASE3 (1:400, Proteintech) and BCL2 (1:200, Proteintech). Briefly, IVD tissue sections were rinsed in PBS and permeabilized in 0.3% Triton X-100 at room temperature for 30 min. The sections were blocked for 1 h in 5% bovine serum albumin (BSA) and 0.1% Triton X-100. The sections were then incubated with antibodies overnight at 4 ^∘^C in a dark, humidified chamber. After washing, the sections were incubated with an Alexa Flu-or-594-conjugated anti-rabbit secondary antibody (Jackson ImmunoResearch) for 60 min. Finally, sections were rinsed with TBST and stained with DAPI for 10 min. Images were visualized at 20× magnification using a DM6B fluorescence microscope (Leica). Immunofluorescence was evaluated using ImageJ software.

### qPCR

The endplates of the IVD were dissected, and roughly 150 mg of NP or AF tissue was gathered. The tissue specimens underwent a one-hour digestion with 2 mg/mL pronase at 37 ^∘^C, followed by rapid freezing, pulverization in liquid nitrogen, and homogenization utilizing a TissueLyser. RNA was isolated with TRIzol (Invitrogen) and reverse-transcribed with SuperScript VILO (Invitrogen). qPCR was conducted on a Real Time System (Bio–Rad). The cycle conditions were: 50 ^∘^C for 2 min, 95 ^∘^C for 2 min, followed by 40 cycles of 15 s at 95 ^∘^C and 1 min at 60 ^∘^C. RPLP0 served as the internal reference gene, and the relative gene expression was determined utilizing the 2^−ΔΔCt^ approach. Primer 6.0 software was used to design primer sequences, which are listed in Table 1. The main selection parameters for screening the primers were as follows: primers should be 18–25 nucleotides long; Tm is between 58 ^∘^C and 62 ^∘^C; GC content of the primers should be between 40% and 60%; avoid having consecutive G or C bases at the 3′ end; qPCR product should be between 100 and 300 bp.

### 1,9-Dimethylmethylene blue (DMMB) analysis

A modified DMMB method was used to measure the cumulative release of GAGs in the IVD culture medium. DMMB solution was prepared by dissolving 16 mg of DMMB, 3.04 g of glycine, and 2.37 g of NaCl in 1 L of distilled water, stirring overnight at room temperature, and covering with tin foil. The pH value of the DMMB solution was adjusted to 3.0 with 1 M HCl (approximately 9.5 mL) so that the absorbance at 525 nm (A525) was 0.31. The solution was stored at room temperature away from light. Then, 20 µL of diluted IVD culture medium was blended with 0.2 mL of DMMB solution, and the A525 was measured instantly with a microplate reader. A series of chondroitin 4-sodium sulfate dilutions was prepared, exhibiting a halving concentration gradient from 125 to 3.90625 µg/mL. The standard GAG reference curve was obtained by mixing 20 µL of diluent with 200 µL of DMMB solution.

### Histomorphological analysis

IVDs were fixed in 4% paraformaldehyde for 48 h at 4 ^∘^C and embedded in O.C.T. (Sakura). Transverse cryosections (10 µm) were cut with a microtome (Leica). The sections were stained with Safranin O-Fast Green (Sigma–Aldrich, S-O) and imaged using a digital pathology system.

### Measurement of IVD height and dynamic compressive stiffness (DCS)

Mean IVD height was measured using a Vernier caliper. Each disc was measured at two separate locations to determine IVD height, and the mean value was normalized to the Day 1 dimension. The height of the IVD measured on Day 1 after overnight culture, before loading, served as the standardized baseline. The percent IVD height change was calculated.

**Figure 2.**
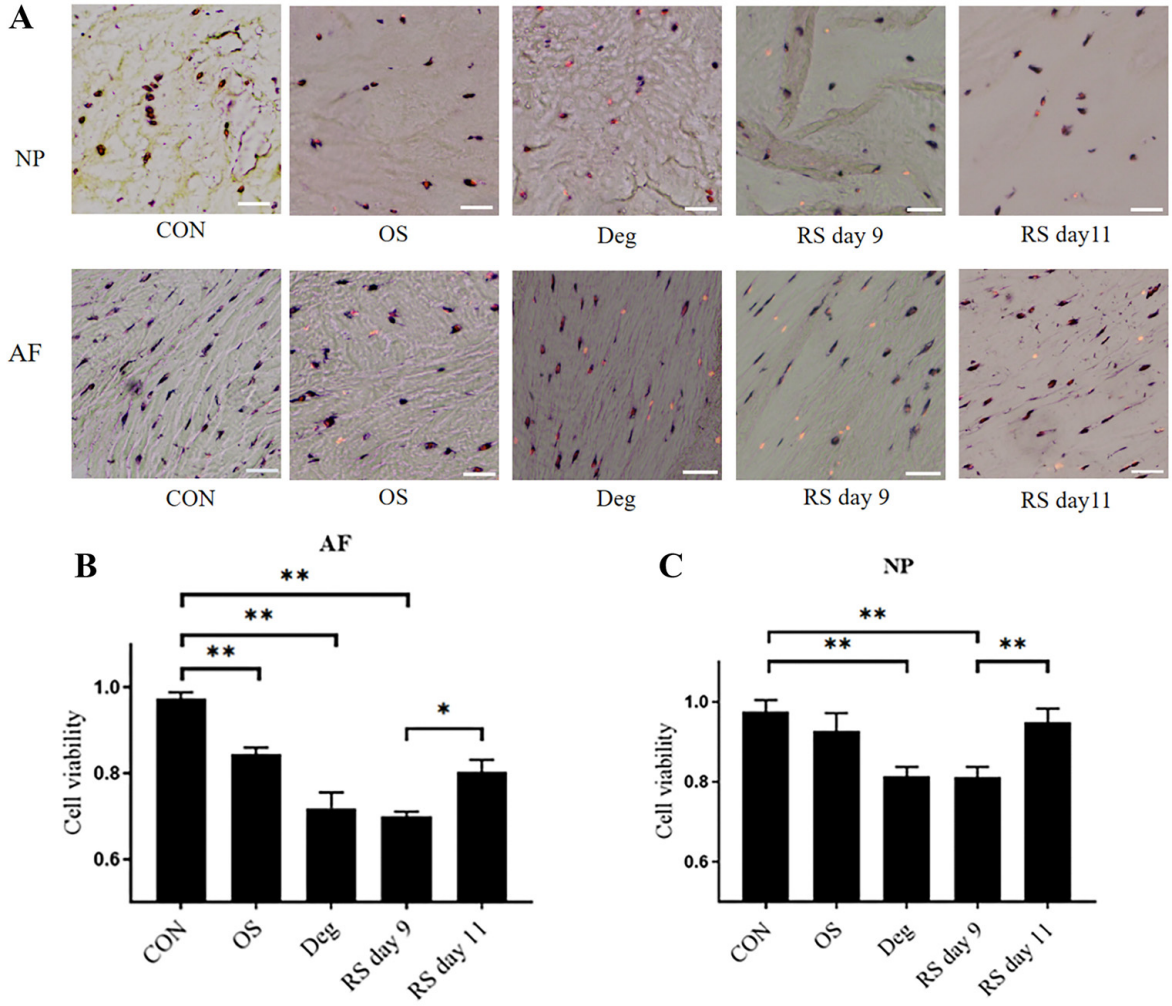
**IVD cell death was induced by strike loading. LDH/EthD-1 staining was employed to assess cell viability.** In the representative images, viable cells appear in blue or blue/red, whereas dead cells are shown in red. CON: Control group on day 11; OS: One strike group on day 11; Deg: Degenerative group on day 11; RS day 9: Repeated strike group measure on day 9; RS day 11: Repeated strike group measure on day 11 (A). Cell viability in the IVD cells is detailed as follows: (B) represents the NP region, and (C) represents the AF region. *n* ═ 4, means ± SEM, Scale bars: 50 µm. *P < 0.05, **P < 0.01. IVD: Intervertebral disc; NP: Nucleus pulposus; AF: Annulus fibrosus.

DCS was measured after overnight culture. The discs were initially subjected to a preloading force of 10 N for 3 min, followed by ten cycles of sinusoidal compression, with strain levels ranging from 5% to 15%. The calculation formula is as follows: (*F*_max_–*F*_min_)/*S*. In this context, *F*_max_ and *F*_min_ represent the peak and nadir forces experienced in each cycle, while S denotes the cross-sectional area of the IVD. Following this, the calculation of the DCS is based on the mean value of 10 rounds. Stiffness measured on Day 1 served as the standardized baseline.

### Statistical analysis

GraphPad Prism 8.0 (San Diego, CA, USA) was used to perform statistical analysis. Data are shown as mean ± SEM. To determine whether the data were normally distributed, data was first checked by the Shapiro–Wilk normality test. The Student’s *t*-test was used for normally distributed data, whereas the Mann–Whitney *U* test was employed for datasets that deviated from a normal distribution. Data yielding a *P* value below 0.05 were regarded as statistically significant.

## Results

### Cell viability

The IVD samples were collected, and frozen sections were prepared for LDH/EthD-1 staining. The images of LDH/EthD-1 staining in each group are shown in (Figure 2A), where blue or blue-red double staining represents live cells, and red staining alone represents dead cells. Compared with the CON group on day 11, the viability of the AF cells in the model groups was significantly decreased (*P* < 0.01), but there was no significant difference among the Deg, OS, and RS groups on day 11. Compared with the RS group on day 11, AF cell viability was further reduced on day 9 (Figure 2B). Deg culture condition and the RS group on day 9 significantly reduced NP cell viability, whereas the RS group on day 11 did not alter NP cell viability (Figure 2C).

### Immunofluorescence

**Figure 3.**
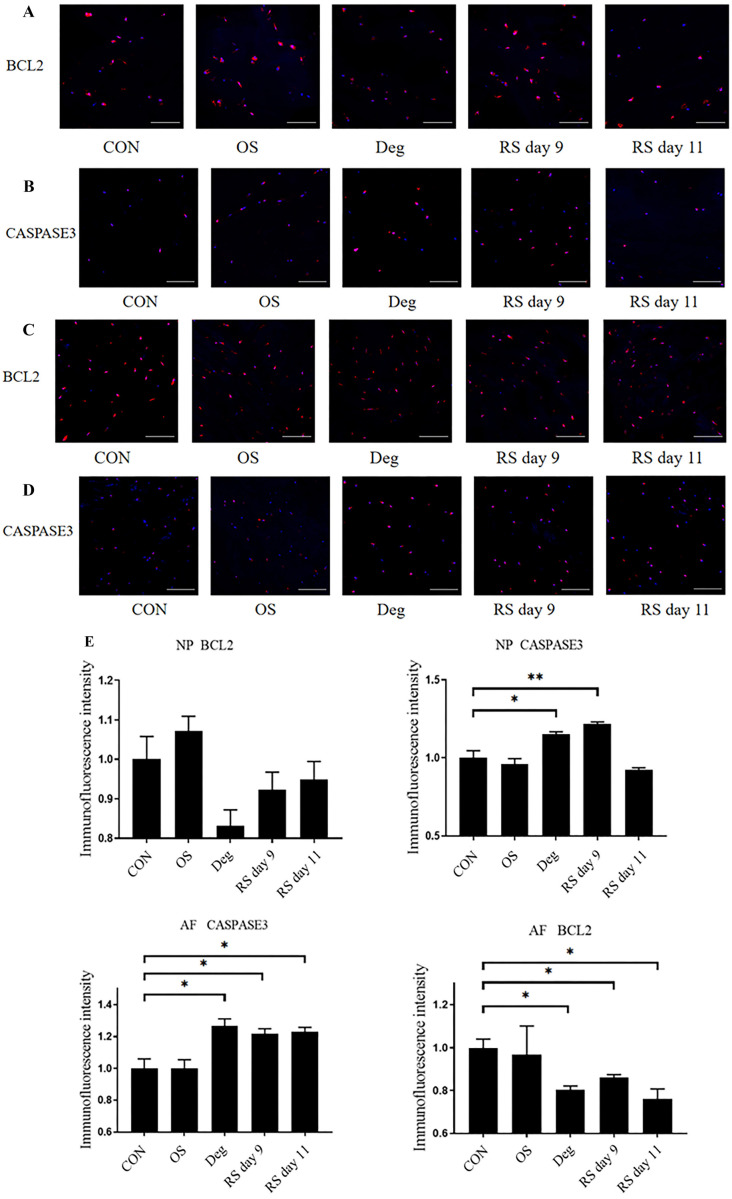
**The expression levels of apoptosis-related proteins were examined using immunofluorescence staining.** (A and B) Represent NP region; (C and D) Represent AF region; (E) Relative expression levels of apoptosis-related proteins. Scale bars: 100 µm. *n* ═ 3, means ± SEM, *P < 0.05, **P < 0.01. CON: Control group on day 11; OS: One strike group on day 11; Deg: Degenerative group on day 11; RS day 9: Repeated strike group measure on day 9; RS day 11: Repeated strike group measure on day 11; NP: Nucleus pulposus; AF: Annulus fibrosus.

Immunofluorescence was used to explore the relationship between cell viability and apoptosis. In NP tissue, Deg culture conditions and RS on day 9 upregulated CASPASE3, and the expression of BCL2 showed a trend of downregulation in the two model groups. In AF tissue, Deg culture conditions and RS downregulated BCL2 and upregulated CASPASE3 (Figure 3A–3E).

**Figure 4.**
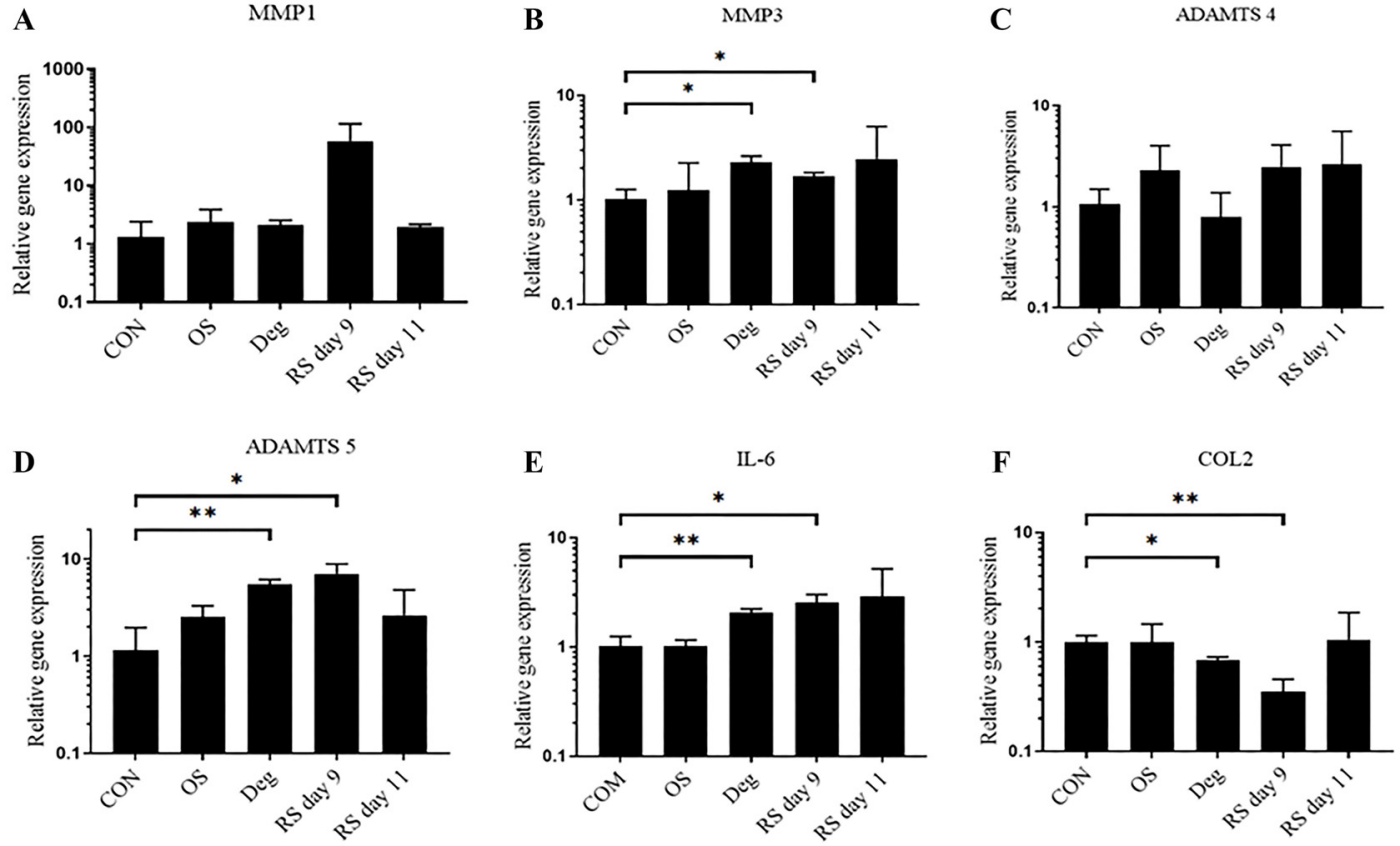
**The gene expression levels in NP tissue were examined using qPCR.** *n* ═ 3, means ± SEM, *P < 0.05, **P < 0.01. CON: Control group on day 11; OS: One strike group on day 11; Deg: Degenerative group on day 11; RS day 9: Repeated strike group measure on day 9; RS day 11: Repeated strike group measure on day 11; NP: Nucleus pulposus.

### Gene expression

In NP tissue, compared with the CON group, the expression levels of the catabolic genes *ADAMTS5* and *MMP3* were significantly upregulated in the Deg group and RS group on day 9. Deg culture conditions and RS on day 9 also down-regulated *COL2* gene expression. Furthermore, the proinflammatory gene expression marker IL-6 was significantly overexpressed in the Deg group and RS group on day 9. However, there was no significant difference observed in the OS group and RS group on day 11 (Figure 4).

**Figure 5.**
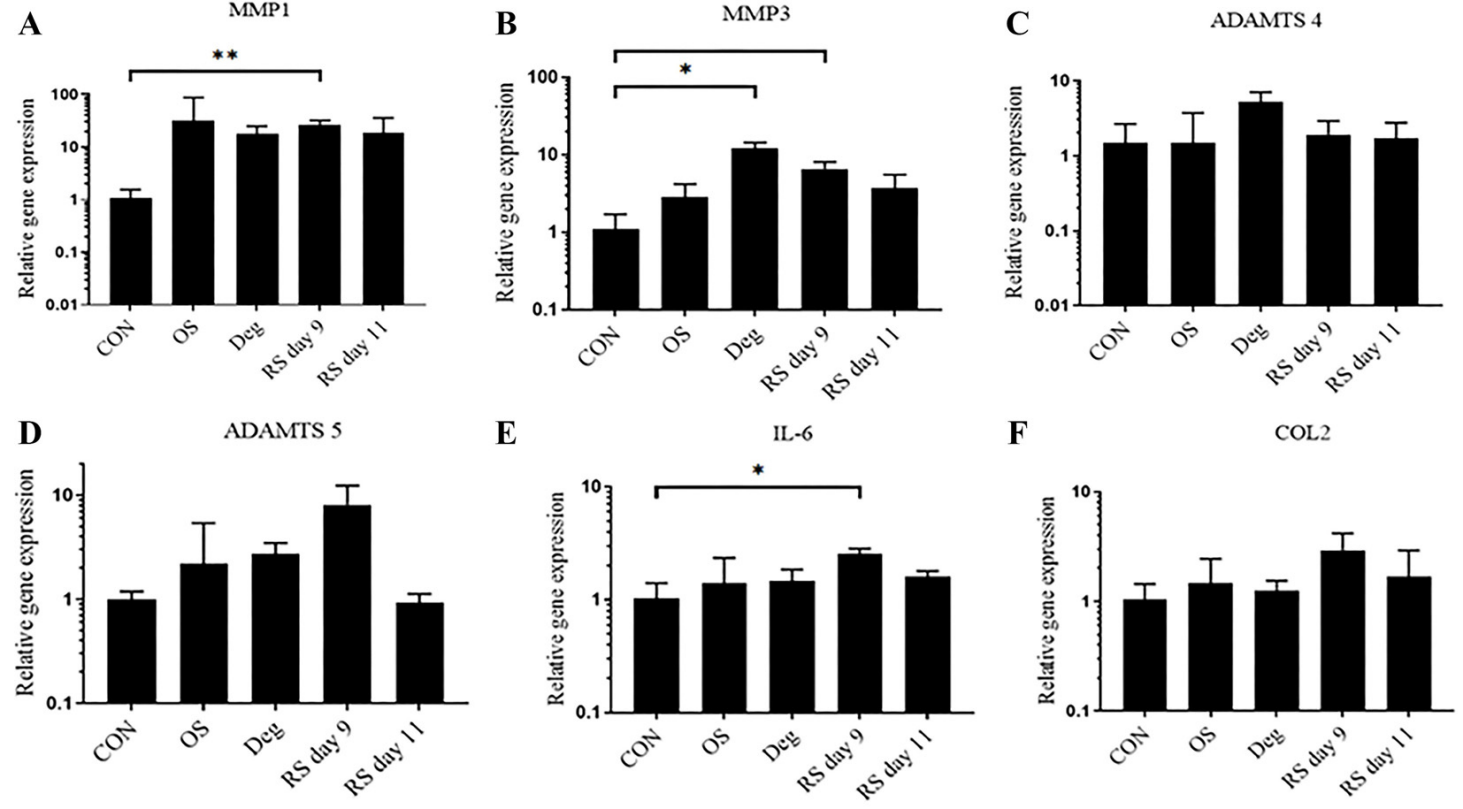
**The gene expression levels in AF tissue were examined using qPCR.** *n* ═ 3, means ± SEM, *P < 0.05, **P < 0.01. CON: Control group on day 11; OS: One strike group on day 11; Deg: Degenerative group on day 11; RS day 9: Repeated strike group measure on day 9; RS day 11: Repeated strike group measure on day 11; AF: Annulus fibrosus.

In AF tissue, the expression levels of the catabolic genes *MMP1* and *MMP3* were significantly upregulated in the RS group on day 9. The gene expression level of *MMP3* was upregulated in the Deg group. The proinflammatory gene *IL-6* was significantly overexpressed in the RS group on day 9. Gene expression remained unaltered in the OS group and RS group on day 11 (Figure 5).

### GAG release

**Figure 6.**
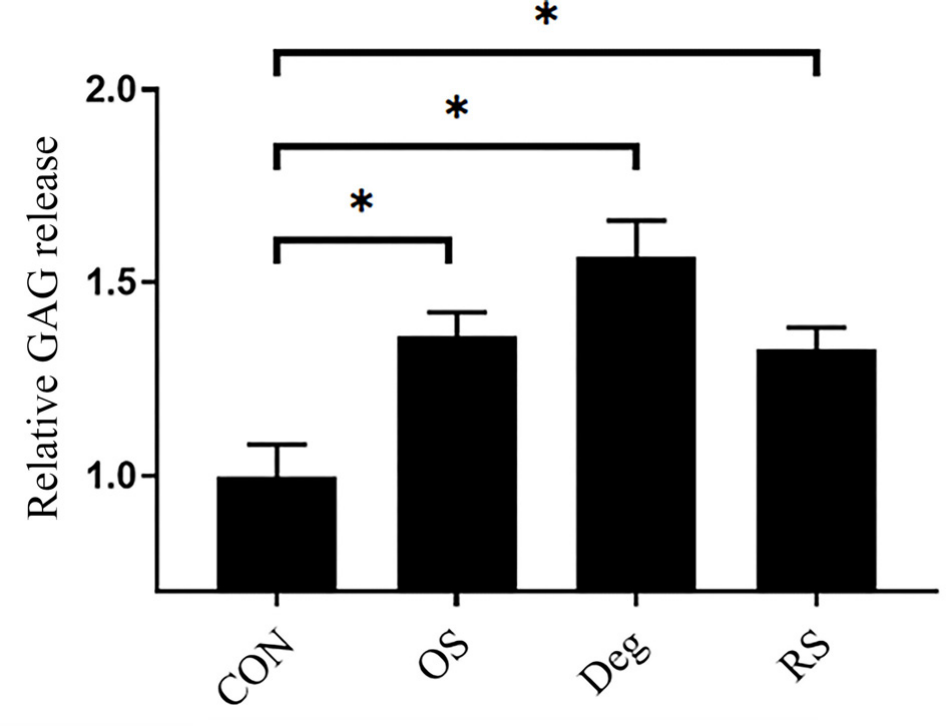
**Relative cumulative release of GAGs into IVD culture medium.** *n* ═ 3, means ± SEM, *P < 0.05. CON: Control group on day 11; OS: One strike group on day 11; Deg: Degenerative group on day 11; RS: Repeated strike group measure on day 11; IVD: Intervertebral disc; GAGs: Glycosaminoglycans.

The DMMB method was used to analyze the cumulative GAG release from the IVDs during the study period. Conditioned medium was collected daily following free-swelling culture and physiological loading culture. The content of released GAG measured on Day 1 following free-swelling culture was used as a baseline for standardization. The DMMB assay demonstrated that GAG release increased in the Deg group and strike loading groups compared with the CON group (*P* < 0.05), but there was no significant difference among the Deg, OS, and RS groups (Figure 6).

### Histomorphological changes

**Figure 7.**
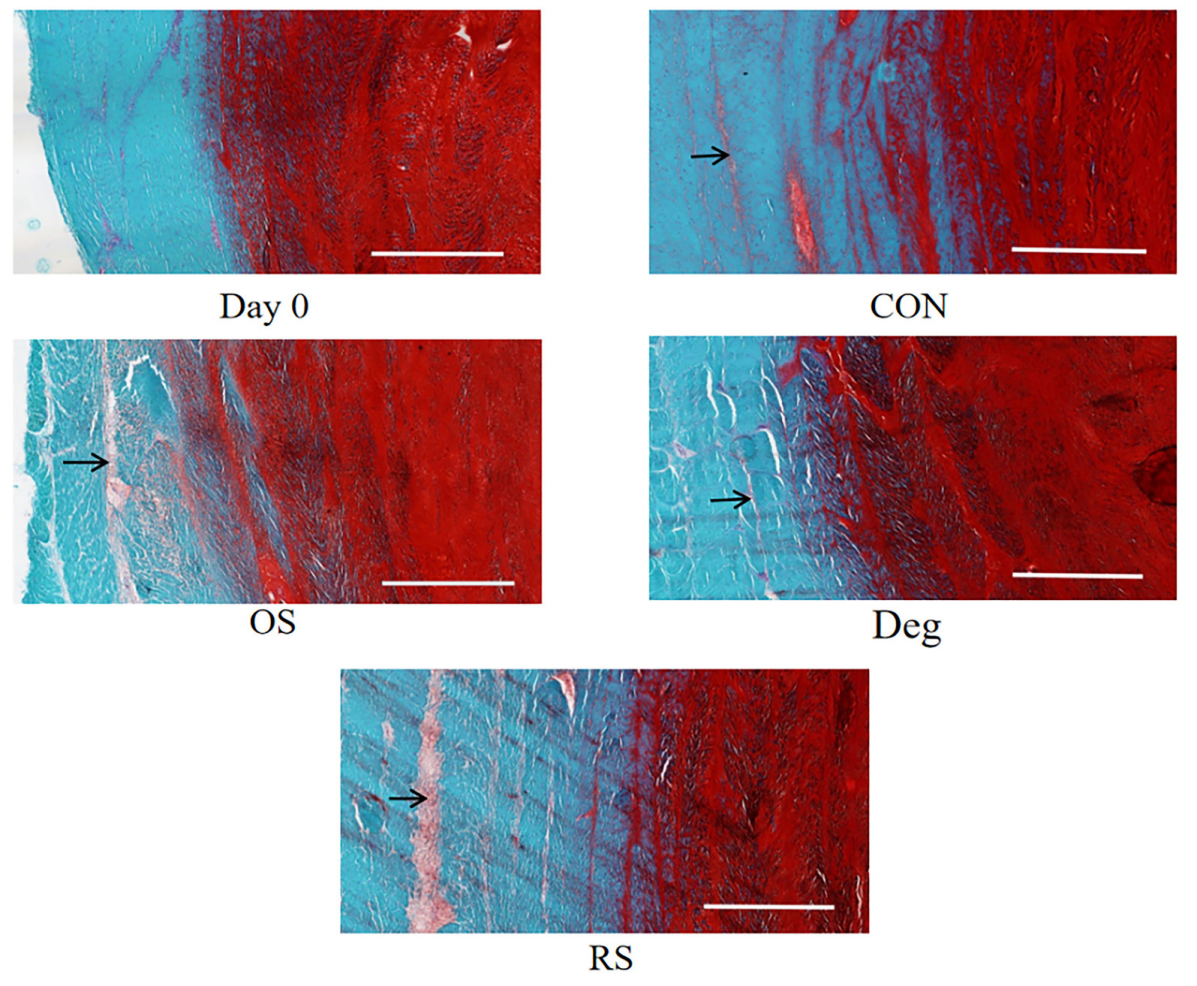
**The microstructure of the IVD was examined using Safranin O-Fast Green.** Black arrow indicates cleft. Day 0: On the day that IVDs were harvested; CON: Control group on day 11; OS: One strike group on day 11; Deg: Degenerative group on day 11; RS: Repeated strike group measure on day 11; IVD: Intervertebral disc.

S-O staining was used to show the morphological changes in the IVD tissue (Figure 7). Compared with the IVD in the Day 0 group, the microstructures of the IVDs in the CON group did not change significantly. In the Day 0 and CON groups, slight to mild clefts were observed in the AF. The majority of the clefts were parallel to the AF lamellae, without anomalous distortions, and little delamination was found. In the Deg, OS, and RS groups, modest and severe clefts were observed in the AF. Additionally, more clefts perpendicular to the AF lamellae were observed. Evident delamination was found in the model groups.

**Figure 8.**
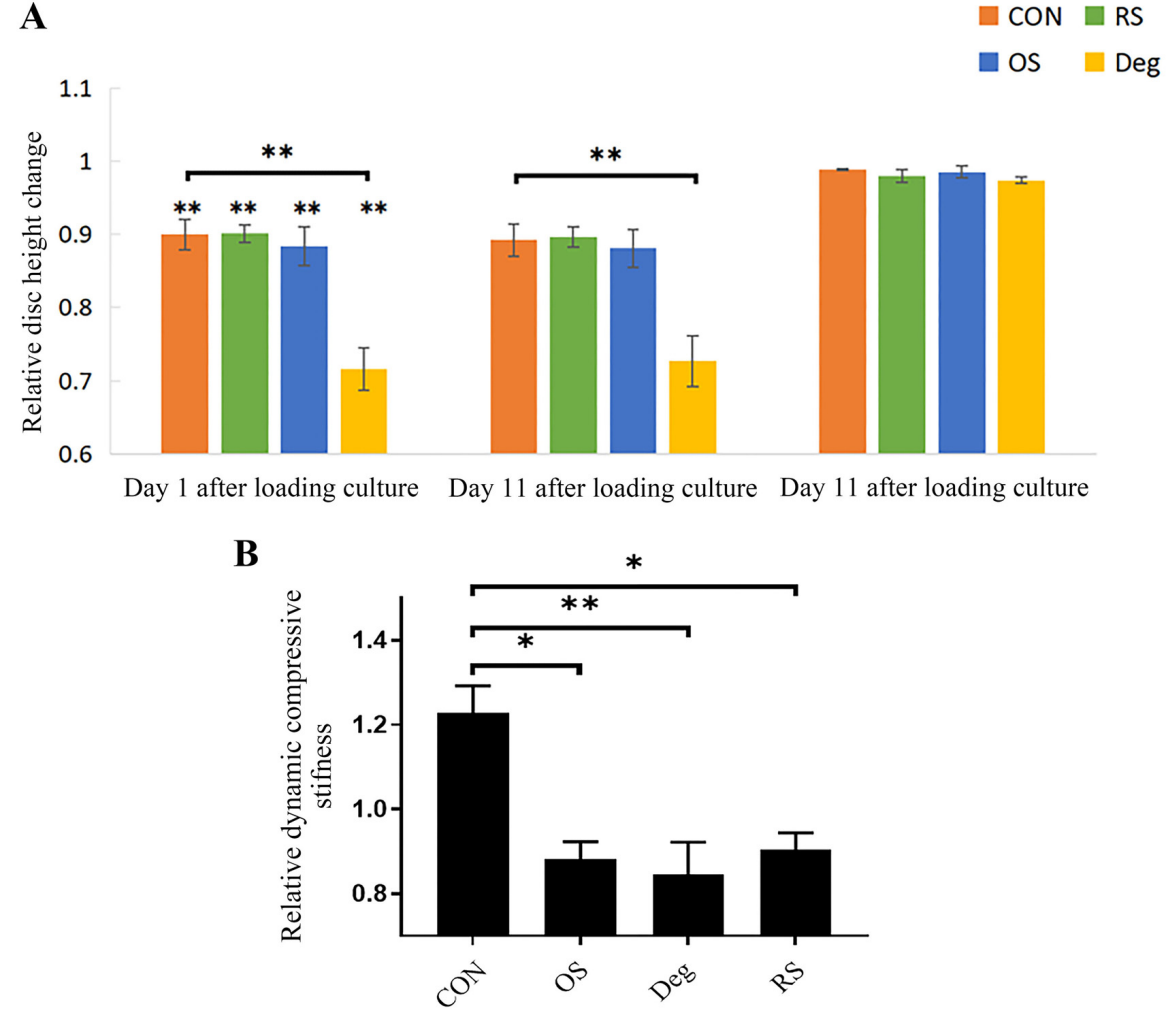
(A) IVD height changes after different culture conditions. In loading culture conditions, IVDs in the CON, OS, and RS groups were measured after physiological loading (20–200 KPa; 0.2 Hz; 1 h). IVDs in the Deg group were measured after degenerative loading (320–500 KPa; 5 Hz; 2 h/day). (B) Changes in the DCS of the IVDs after 11 days of culture. DCS measured on Day 1 served as the standardized baseline. *n* ═ 3, means ± SEM, *P < 0.05, **P < 0.01. CON: Control group; OS: One strike group; Deg: Degenerative group; RS: Repeated strike group measure; IVD: Intervertebral disc; DCS: Dynamic compressive stiffness.

### IVD height and DCS

The results showed that the height of the IVD significantly decreased in all groups after physiological loading culture on day 1 (*P* < 0.01). On Day 11, all IVD heights showed no significant difference compared to that on Day 1 after overnight free-swelling culture (Figure 8A).

After 11 days of culture, the IVDs in the CON group showed an increasing trend in DCS compared to day 1, but there was no significant difference (*P* > 0.05). The IVDs in the Deg, OS, and RS groups showed a significant decrease in DCS compared to the CON group (*P* < 0.05), but there was no significant difference among the Deg, OS, and RS groups (*P* > 0.05) (Figure 8B).

## Discussion

This study is based on previous work in which we cultured bovine IVDs in a whole organ culture system with physiological loading or one strike loading. In that study, cell viability was maintained for ten days after subjection to physiological loading. Although strike loading alone caused a substantial reduction in cell viability and gene expression changes in the short term, several of these degradation changes were abolished in the long-term experiment [22]. The goal of the present study was to explore whether repeated strike loading could induce persistent IVD degeneration. Our results indicate that repeated strike loading, one strike loading, and degenerative loading culture could lead to IVD degeneration, such as decreased cell viability, increased release of GAGs, decreased DCS, and matrix structure destruction. Repeated strike loading has a long-term effect on the catabolic and apoptotic gene expression of AF cells. In response to mechanical stress damage, NP cells demonstrate a rapid recovery to their normal state, highlighting their robust adaptability and resilience. In contrast, AF cells exhibit a more prolonged response to mechanical stress, characterized primarily by cell decreased viability and alterations in gene expression. Overall, these findings reveal different responses of NP and AF cells to mechanical stress injury, highlighting AF cells as a potentially more vulnerable and critical element in IVD degeneration and damage.

Our study corroborates previous research on the adverse effects of mechanical stress on IDD. Croft et al. [23] demonstrated that more intense dynamic loading regimes lead to more pronounced IDD levels. Dudli et al. [24] found that endplate fractures significantly promote disc degeneration, while impact loading without fractures causes minimal changes in cell viability, apoptosis, and inflammation markers. Illien-Junger et al. [25] examined high-frequency loading under various nutritional conditions over seven days, emphasizing the harmful effects of combined mechanical and nutritional stress. While their study highlighted the detrimental effects of combined mechanical and nutritional stress, our study emphasizes the role of repeated mechanical stress over a longer duration, highlighting the cumulative effects on disc degeneration. Zhou et al. [22], using a one-strike loading model, observed early degenerative changes post-trauma. Our repeated strike loading model, however, provides additional insights into how chronic repetitive loading further exacerbates these degenerative processes over time. Haschtmann et al. [26] indicated that a single hyperphysiological compression event causes endplate fractures and significant cell death. In contrast, our repeated strike loading model shows the cumulative impact of mechanical stress on disc degeneration, with significant increases in apoptosis, matrix remodeling, and pro-inflammatory responses. These findings highlight the critical role of repetitive mechanical stress in accelerating degenerative processes and emphasize the importance of considering the removal of mechanical loads in preventing and treating IDD.

At present, experimental studies on the relationship between abnormal mechanical stress and IVD degeneration mostly use chronic sustained mechanical injury or acute violent injury models [27–29]. Chronic sustained mechanical injury can lead to persistent IVD degeneration, while acute abnormal mechanical stress injury can induce transient and short-term IVD degeneration [30]. Previous investigations have identified that impact loading can precipitate degeneration within the IVDs [22, 28, 31]. However, within such models, significant disruptions occur, including the destruction of disc endplates, tears in the AF, and alterations to the low oxygen and nutrient environments in the NP, alongside changes in mechanical characteristics. It remains uncertain whether impact loading results in irreversible damage to the IVD, rendering it incapable of recovery. Consequently, these models may be unsuitable for the study of early-stage IVD degeneration. Another study using the dynamic compression model of disc degeneration incorporates factors of nutritional limitation, unsuited to accurately assess the impact of mechanical stress on disc degeneration [25]. Additionally, the model necessitates daily compression-induced damage, diverging from the natural human response to disc injury, thereby limiting the observation of therapeutic effects on discs following the cessation of injury.

There are few studies on the effects of repeated injuries on IVD degeneration. Ulrich et al. [18] used a No. 11 surgical blade to puncture and damage rat caudal IVDs on days 1, 3, and 6 of the experiment and found that repeated injuries resulted in a continuous inflammatory response and persistent degeneration of IVDs. As the integrity of the IVD becomes damaged, the NP loses its protective isolation environment and triggers a molecular cascade immune response. Therefore, this model is not suitable to explore the early impact of repeated injury on IVD degeneration. In this study, we used the strike-loading IVD injury model from previous reports and performed repeated strike-loading injuries to the IVD [19, 22]. Since the heights of the IVDs in each group did not change significantly after swelling, the strike-loading IVD degeneration model used in this experiment has maintained relatively normal mechanical properties. This disc degeneration model induced by strike-loading is suitable for studying the early stages of disc degeneration, especially in the absence of significant structural destruction. In the study of IVDs with severe degeneration, the endplate or AF of the IVD organ model should be destroyed to simulate the structural defect and mechanical instability of the disc during severe IDD.

In the context of IVD degeneration, it is observed that a reduction in hydration is a concomitant phenomenon of the process, contrasting with the high hydration state inherent to a healthy disc [32]. The water content in healthy NP tissue can be as high as 90%, and in AF tissue, it is approximately 65%–70% [33, 34]. The difference in GAG content is the main reason for the difference in water content between the two tissues. GAGs are negatively charged and have a fixed charge density, attracting positive charges into the NP matrix [35]. Therefore, the NP can absorb much more water than its weight and lock the water molecules through polar interactions in the tissue, which makes the IVD in vitro culture have a strong tendency to swell and cause swelling stress. During the disc culture cycle in this study, neither mechanical stress injury mode resulted in disc height reduction after swelling culture. Since GAGs are primarily located in the center of the IVD, the narrowing of the space caused by the catabolism of the small amount of GAGs is filled by additional water absorbed by the remaining GAGs, resulting in no significant change in IVD height. Croft et al. [23] also employed a bovine organ culture model with a bioreactor and reported a significant loss of relative IVD height in the model groups after one week of loading. They viewed IVD height loss as a sign of IDD.

These inferences are supported by the DCS of the discs. Measurements of the DCS can indirectly reflect the changes in GAG content in tissues [36]. The IVD GAG content is directly correlated with tissue swelling stress. As the amount of IVD proteoglycan decreases with age and degeneration, the total fixed charge density of the tissue decreases, leading to a decrease in water retention. The swelling stress of degenerative discs was significantly lower than that of normal disc tissue [36]. In this experiment, we measured the DCS of the disc organ. DCS is a measure of the average amount of stress generated by the disc in the 10% range of deformation. The results showed that both mechanical stress injury modes reduced the DCS of the discs. The dynamic stiffness of the physiological CON group on the 11th day increased compared to that on the 1st day. This may be related to the disc organ absorbing more water in the in vitro medium, leading to an increase in swelling stress and subsequently an increase in dynamic stiffness.

There are several limitations that should be highlighted in the present research. First, the disc organ culture model did not incorporate blood vessels or the immune system, both critical components in disc degeneration processes. The absence of a vascular and immune response in organ culture models underscores the necessity for a multifaceted research approach to fully elucidate the mechanisms of disc degeneration. Second, the structural integrity of the IVD is preserved, with the NP remaining encapsulated within the AF. This model, both from a mechanical and biological perspective, is specifically suited for investigating responses associated with the early stages of IVD degeneration. Additionally, despite their applicability in simulating IDD, bovine tail and human IVDs differ significantly in anatomy, biomechanics, cellular composition, and degeneration manifestations. These differences must be considered when using bovine discs as models. Finally, the IVDs were not subjected to extended culturing periods, precluding the exploration of prolonged degenerative mechanisms. Future research could benefit from focusing on long-term culture studies to elucidate the progressive degenerative processes and explore potential regenerative therapies.

## Conclusion

In conclusion, our results provide evidence that repeated strike loading could induce degenerative changes in the IVD. As continuous mechanical stress is required to induce long-lasting molecular changes, repeated strike loading is apt for studying chronic IDD. The results also indicated that alleviating abnormal mechanical conditions is a very effective way to halt or even reverse IDD progression. Additionally, we found that the AF is more susceptible to mechanical stress, which may provide a new perspective on IDD research.

## References

[1] Mohd Isa IL, Teoh SL, Mohd Nor NH, Mokhtar SA (2022). Discogenic low back pain: anatomy, pathophysiology and treatments of intervertebral disc degeneration. Int J Mol Sci.

[2] Alonso-Garcia M, Sarria-Santamera A (2020). The economic and social burden of low back pain in Spain: a national assessment of the economic and social impact of low back pain in Spain. Spine (Phila Pa 1976).

[3] Wu X, Liu W, Duan Z, Gao Y, Li S, Wang K (2016). The involvement of protease Nexin-1 (PN1) in the pathogenesis of intervertebral disc (IVD) degeneration. Sci Rep.

[4] He F, Chen Z, Su Q, Yan M, Zhang Q, Tan J (2019). Melatonin modulates IL-1beta-induced extracellular matrix remodeling in human nucleus pulposus cells and attenuates rat intervertebral disc degeneration and inflammation. Aging (Albany, NY),.

[5] Desmoulin GT, Pradhan V, Milner TE (2020). Mechanical aspects of intervertebral disc injury and implications on biomechanics. Spine (Phila Pa 1976).

[6] Cassidy JJ, Hiltner A, Baer E (1989). Hierarchical structure of the intervertebral disc. Connect Tissue Res.

[7] Ohnishi T, Novais EJ, Risbud MV (2020). Alterations in ECM signature underscore multiple sub-phenotypes of intervertebral disc degeneration. Matrix Biol Plus.

[8] Inoue N, Espinoza Orias AA (2011). Biomechanics of intervertebral disk degeneration. Orthop Clin North Am.

[9] Li X, Liu Y, Li L, Huo R, Ghezelbash F, Ma Z (2023). Tissue-mimetic hybrid bioadhesives for intervertebral disc repair. Mater Horiz.

[10] Alini M, Eisenstein SM, Ito K, Little C, Kettler AA, Masuda K (2008). Are animal models useful for studying human disc disorders/degeneration?. Eur Spine J.

[11] Lotz JC (2004). Animal models of intervertebral disc degeneration: lessons learned. Spine (Phila Pa 1976).

[12] Calio M, Gantenbein B, Egli M, Poveda L, Ille F (2021). The cellular composition of bovine coccygeal intervertebral discs: a comprehensive single-cell RNAseq Analysis. Int J Mol Sci.

[13] Adam C, Rouch P, Skalli W (2015). Inter-lamellar shear resistance confers compressive stiffness in the intervertebral disc: an image-based modelling study on the bovine caudal disc. J Biomech.

[14] Hwang D, Gabai AS, Yu M, Yew AG, Hsieh AH (2012). Role of load history in intervertebral disc mechanics and intradiscal pressure generation. Biomech Model Mechanobiol.

[15] Dunn IF, Proctor MR, Day AL (2006). Lumbar spine injuries in athletes. Neurosurg Focus.

[16] Chen B, Grazi L, Lanotte F, Vitiello N, Crea S (2018). A real-time lift detection strategy for a hip exoskeleton. Front Neurorobot.

[17] Bai X, Wang D, Zhou M, Xu C, Li W, Tao H (2017). Noninvasive cumulative axial load may induce intervertebral disc degeneration-A potential rabbit model. Exp Ther Med.

[18] Ulrich JA, Liebenberg EC, Thuillier DU, Lotz JC (2007). ISSLS prize winner: repeated disc injury causes persistent inflammation. Spine (Phila Pa 1976).

[19] Li BL, Liu X, Gao M, Zhang F, Chen X, He Z (2021). Programmed NP cell death induced by mitochondrial ROS in a one-strike loading disc degeneration organ culture model. Oxid Med Cell Longev.

[20] Lang G, Liu Y, Geries J, Zhou Z, Kubosch D, Südkamp N, Richards RG (2018). An intervertebral disc whole organ culture system to investigate proinflammatory and degenerative disc disease condition. J Tissue Eng Regen Med.

[21] Stoddart MJ, Furlong PI, Simpson A, Davies CM, Richards RG (2006). A comparison of non-radioactive methods for assessing viability in ex vivo cultured cancellous bone: technical note. Eur Cell Mater.

[22] Zhou Z, Cui S, Jie D, Richards RG, Alini M, Grad S (2021). One strike loading organ culture model to investigate the post-traumatic disc degenerative condition. J Orthop Translat.

[23] Croft AS, Roth Y, Oswald KAC, Corluka S, Bermudez-Lekerika P, Gantenbein B (2021). In situ cell signalling of the hippo-YAP/TAZ pathway in reaction to complex dynamic loading in an intervertebral disc organ culture. Int J Mol Sci.

[24] Dudli S, Haschtmann D, Ferguson SJ (2012). Fracture of the vertebral endplates, but not equienergetic impact load, promotes disc degeneration in vitro. J Orthop Res.

[25] Illien-Junger S, Gantenbein-Ritter B, Grad S, Lezuo P, Ferguson SJ, Alini M (2010). The combined effects of limited nutrition and high-frequency loading on intervertebral discs with endplates. Spine (Phila Pa 1976).

[26] Haschtmann D, Stoyanov JV, Gédet P, Ferguson SJ (2008). Vertebral endplate trauma induces disc cell apoptosis and promotes organ degeneration in vitro. Eur Spine J.

[27] Ching CCT, Chow DHK, Yao FYD, Holmes AD (2003). The effect of cyclic compression on the mechanical properties of the inter-vertebral disc: an in vivo study in a rat tail model. Clin Biomech (Bristol, Avon).

[28] Dudli S, Haschtmann D, Ferguson SJ (2015). Persistent degenerative changes in the intervertebral disc after burst fracture in an in vitro model mimicking physiological post-traumatic conditions. Eur Spine J.

[29] Wuertz K, Godburn K, MacLean JJ, Barbir A, Donnelly JS, Roughley PJ (2009). In vivo remodeling of intervertebral discs in response to short- and long-term dynamic compression. J Orthop Res.

[30] Lotz JC, Ulrich JA (2006). Innervation, inflammation, and hypermobility may characterize pathologic disc degeneration: review of animal model data. J Bone Joint Surg Am.

[31] Alkhatib B, Rosenzweig DH, Krock E, Roughley PJ, Beckman L, Steffen T (2014). Acute mechanical injury of the human intervertebral disc: link to degeneration and pain. Eur Cell Mater.

[32] Wang D, Li Z, Huang W, Cao S, Xie L, Chen Y (2023). Single-cell transcriptomics reveals heterogeneity and intercellular crosstalk in human intervertebral disc degeneration. iScience.

[33] Brissenden AJ, Amsden BG (2023). In situ forming macroporous biohybrid hydrogel for nucleus pulposus cell delivery. Acta Biomater.

[34] Wang Z, Chen X, Chen N, Yan H, Wu K, Li J (2024). Mechanical factors regulate annulus fibrosus (AF) injury repair and remodeling: a review. ACS Biomater Sci Eng.

[35] Silagi ES, Shapiro IM, Risbud MV (2018). Glycosaminoglycan synthesis in the nucleus pulposus: Dysregulation and the pathogenesis of disc degeneration. Matrix Biol.

[36] Yang B, O’Connell GD (2019). Intervertebral disc swelling maintains strain homeostasis throughout the annulus fibrosus: a finite element analysis of healthy and degenerated discs. Acta Biomater.

